# Video Surveillance Captures Student Hand Hygiene Behavior, Reactivity to Observation, and Peer Influence in Kenyan Primary Schools

**DOI:** 10.1371/journal.pone.0092571

**Published:** 2014-03-27

**Authors:** Amy J. Pickering, Annalise G. Blum, Robert F. Breiman, Pavani K. Ram, Jennifer Davis

**Affiliations:** 1 Woods Institute for the Environment, Stanford University, Stanford, California, United States of America; 2 Environment and Water Studies, Civil and Environmental Engineering, Stanford University, Stanford, California, United States of America; 3 Global Disease Detection Program, Kenya Office of the Centers for Disease Control and Prevention, Nairobi, Kenya; 4 Social and Preventative Medicine, State University of New York at Buffalo, Buffalo, New York, United States of America; 5 Global Disease Detection Branch, Division of Global Health Protection, Center for Global Health, Centers for Disease Control and Prevention, Atlanta, Georgia, United States of America; University of Pittsburgh Medical Center, United States of America

## Abstract

**Background:**

In-person structured observation is considered the best approach for measuring hand hygiene behavior, yet is expensive, time consuming, and may alter behavior. Video surveillance could be a useful tool for objectively monitoring hand hygiene behavior if validated against current methods.

**Methods:**

Student hand cleaning behavior was monitored with video surveillance and in-person structured observation, both simultaneously and separately, at four primary schools in urban Kenya over a study period of 8 weeks.

**Findings:**

Video surveillance and in-person observation captured similar rates of hand cleaning (absolute difference <5%, p = 0.74). Video surveillance documented higher hand cleaning rates (71%) when at least one other person was present at the hand cleaning station, compared to when a student was alone (48%; rate ratio  = 1.14 [95% CI 1.01–1.28]). Students increased hand cleaning rates during simultaneous video and in-person monitoring as compared to single-method monitoring, suggesting reactivity to each method of monitoring. This trend was documented at schools receiving a handwashing with soap intervention, but not at schools receiving a sanitizer intervention.

**Conclusion:**

Video surveillance of hand hygiene behavior yields results comparable to in-person observation among schools in a resource-constrained setting. Video surveillance also has certain advantages over in-person observation, including rapid data processing and the capability to capture new behavioral insights. Peer influence can significantly improve student hand cleaning behavior and, when possible, should be exploited in the design and implementation of school hand hygiene programs.

## Introduction

Hand hygiene promotion programs are increasingly common around the world. Measurement of hand hygiene behavior is important for evaluating program effectiveness, as well as for understanding the relationship between hygiene and health. Few methods exist, however, to measure hand hygiene behavior reliably, accurately, and efficiently [1]. One of the most commonly used and least costly methods is to obtain self-reported data through in-person interviews. Such data are often biased by social desirability effects, particularly when respondents are participating in a hand hygiene intervention [2], [3]. Rapid observation (i.e., spot checks) of the presence and location of hand cleaning supplies (*e.g*. soap, water) has been shown to correlate with handwashing behavior [4]. Motion sensors placed inside bars of soap have also been used to measure use[5]. Rapid observations and sensors provide no information, however, about which household member is using the soap, whether it is being used for handwashing exclusively, or whether the soap is used at critical times, such as after using the toilet or before feeding a child. In-person structured observation, in which a human observer spends several hours watching and documenting a subject's behavior, is often considered the gold standard for measuring hand hygiene behavior. Observation is time consuming and expensive relative to other data collection strategies, however, and has been shown to alter subjects' behavior[5]–[9].

Some studies have used video surveillance to capture hand hygiene behavior in high-income countries. Video cameras were used to capture hand hygiene behavior in hospitals in the United States, Japan, and China as early as the 1990s [10]–[12]. A more recent study of health care workers in a United States intensive care unit found video-recorded rates of hand cleaning to be less than 10%, as compared to rates of 60% as measured during in-person observation conducted in the same location[13]. Video monitoring of hand hygiene has also been used outside hospitals to measure hand cleaning at a petting zoo in Canada[14] and to monitor food hygiene in Australian kitchens[15]. However, video monitoring of hand hygiene has yet to be validated against in-person structured observation, or employed to monitor hand hygiene behavior in low-income settings.

The main objective of this analysis was to evaluate the viability of using video cameras to measure hand-cleaning behavior accurately within a low-income school setting. At primary schools in Kenya, video cameras recorded footage of hand cleaning stations located next to latrines. We compare data on hand cleaning rates after toileting events captured by video camera with data collected through in-person structured observation to assess concordance between the two methods. We also explore student reactivity to video surveillance and in-person observation by assessing rates of hand cleaning during simultaneous monitoring *versus* rates captured by each method independently. In addition, by comparing rates of hand cleaning when students are alone *versus* in the presence of others, we assess peer influences on hand hygiene behavior. Finally, the benefits and drawbacks of using cameras to measure hand hygiene behavior are discussed.

## Methods

This study was conducted in four primary schools located within the informal settlement of Kibera, located in Nairobi, Kenya. Data collection occurred between September and November 2010. The four schools were participating in a school-based hand hygiene evaluation of alcohol-based hand sanitizer compared to traditional soap; schools were selected based on similar student populations, water supply, and sanitation characteristics. The full hand hygiene study design is described elsewhere[16]. Eligible schools had at least 100 enrolled students and latrines located on the school premises. Two of the schools were randomly assigned to receive a hand sanitizer intervention, and the other two schools assigned to receive a hand washing with soap and water intervention. A wall dispenser filled with hand sanitizer or liquid hand soap (depending on intervention assignment) was installed outside the latrines at each school. Schools assigned to the soap and water intervention were also provided with water tanks, but were responsible for obtaining water to fill the tanks.

**Figure 1:**
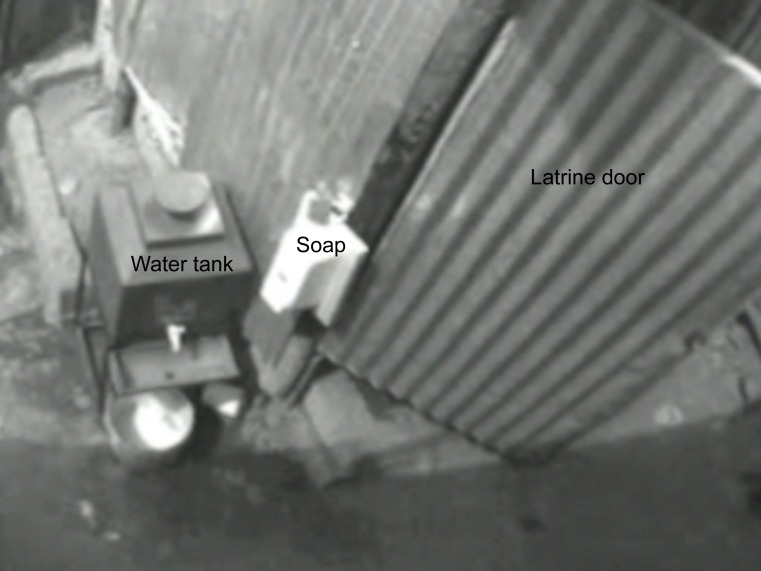
Still frame from a camera positioned above a handwashing with soap station next to a latrine (door is open).

**Figure 2:**
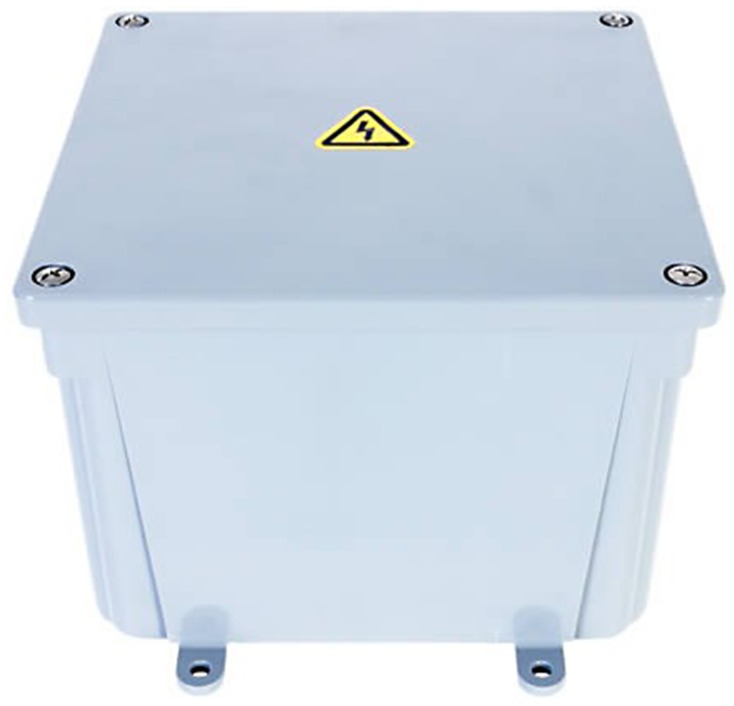
Electrical box camera case; camera lens is concealed by yellow electrical sticker in center (image courtesy of Supercircuits, Inc.).

Video cameras were positioned such that the camera surveillance frames included the doors to the latrines as well as the hand cleaning station (Figure 1). The cameras had motion sensors to initiate recording when any movement was detected within the frame, such as when students entered or exited latrines and/or used the hand cleaning stations. Over a period of 8 weeks, cameras were placed at each school 2–4 days per week to record between the hours of 8:30am and 4:00pm. At the end of each recording day, the cameras were removed to download footage files and recharge the batteries. The cameras were enclosed in plastic electrical boxes (Supercircuits, Inc. Austin, TX), mounted on wooden boards, and padlocked in place. The electrical box was designed to conceal the presence of the enclosed camera (Figure 2); however, students were informed about the presence of the cameras by school staff.

**Figure 3:**
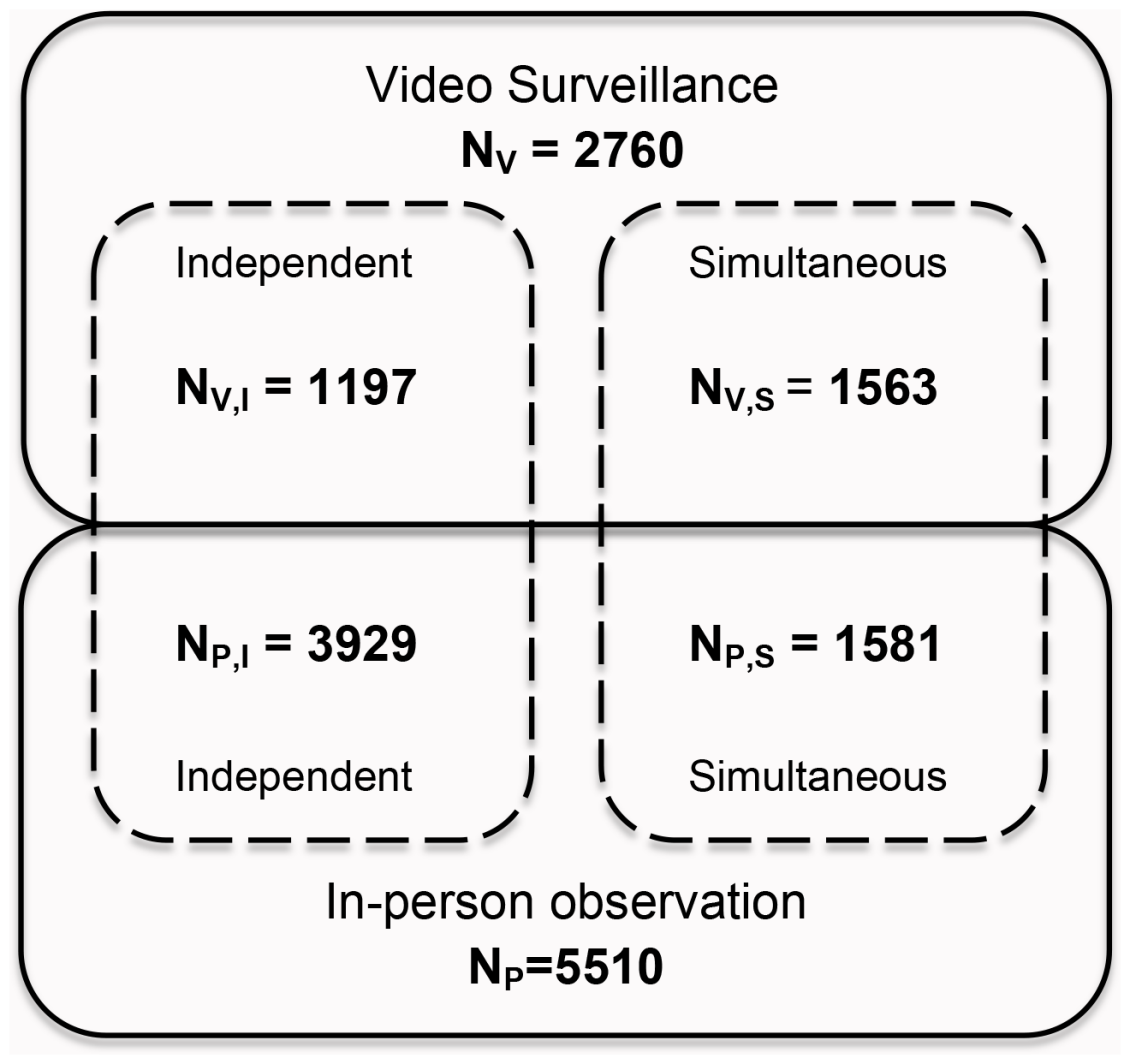
Schematic of study design. Number of toileting events captured on video surveillance (N_V_) and through in-person observation (N_P_), during independent (single-method) *versus* simultaneous monitoring periods.

A rotating field schedule was employed to capture toileting events simultaneously and separately by video surveillance and in-person observation (Figure 3). During a subset of video recording days, in-person observation was conducted concurrently by trained Kenyan enumerators between the hours of 10:30am and 1:30pm. In-person observation was also conducted on days that video cameras were not recording, to enable comparison of hygiene behavior that was captured by in-person observation alone (N_P,I_, Figure 3), video surveillance alone (N_V,I_), and simultaneous in-person observation and video surveillance (N_P,S_ and N_V,S_). In addition, on days with simultaneous monitoring, video cameras recorded from 9:30am–10:30am, the hour preceding the start of in-person observation.

To assess teacher awareness of student reactivity to observation, in-depth interviews were conducted with 3 teachers per school (N = 12). These semi-structured interviews included open-ended questions about whether and how the CCTVs and in-person observation affected hand hygiene behavior at the school. The interviews were voice recorded, transcribed, and then translated into English.

In-person observation and video surveillance coding were generated with the use of personal digital assistants (PDAs) loaded with a survey instrument programmed in The Survey System (Creative Research Systems, Petaluma, CA). Trained observers sat within view of the latrines during in-person observation and coded toileting events in real time using PDAs. Observers did not initiate interaction with students or teachers during data collection. Video footage was coded by 4 research assistants at Stanford University. Surveillance footage without students in the camera frame was viewed at increased speed using the fast-forward feature in media viewing software. When a student subject appeared in the frame, the footage was typically observed in real time, paused if necessary, and rewound to capture details if needed. Qualitative notes were also recorded to document relevant details and behaviors not captured by the survey instrument on the PDA.

For both in-person observation and video surveillance, each student exit from a latrine was considered a “toileting event.” When multiple students cleaned their hands simultaneously, both in-person and video coders were instructed to select one student arbitrarily to follow. Observers recorded if a student cleaned his or her hands with water, soap, and/or sanitizer following each toileting event. Observers also documented the student's gender (by the school uniform), the duration of hand cleaning in seconds, and the method of drying hands. Video coders also collected data on the number of other students visible within the camera frame.

### Data analysis

In-person observation and video footage data for each school were compared in aggregate across all observations. The difference in rates captured by the two methods (rate ratios) of any type of hand cleaning and hand cleaning with product were estimated using Poisson regression, including binary variables to identify the individual school at which the data were collected (school fixed effects). The modeling results were also stratified by intervention type. In-person observation and video footage data were then matched by concurrent 3-hour time blocks. A date and time stamp of each toileting event was recorded automatically by the PDA during in-person observations, and by the camera itself for the video recordings. To compare in-person structured observation with video observation on the same day, video data were coded as occurring on a day without in-person observation or on a day with in-person observation. The number of toileting and hand cleaning events was summed within each of these periods, then merged to obtain a complete data set for each school day. For these simultaneous observation periods, independent sample t-tests were used to assess significant differences in overall hand cleaning rates, hand cleaning rates with soap, and the number of toileting events captured by each method.

To explore reactivity to video surveillance, hand cleaning rates captured by in-person observation were compared between days with and without concurrent video camera surveillance. A similar analysis was conducted with video surveillance data to estimate reactivity to structured observation. Rate ratios (between independent and simultaneous monitoring) were calculated using Poisson regression, controlling for the individual school the data were collected from. Modeling results were also stratified by intervention type to examine any differences in reactivity between sanitizer and soap intervention schools. In addition, on days with simultaneous monitoring, hand cleaning rates captured by video surveillance in the hour preceding in-person observation were compared to rates captured once in-person observation had begun.

### Ethics Statement

Administrators from the study schools gave written consent for video cameras to be placed in public locations on the school premises. Written consent to observation was also obtained from teachers and the parents of enrolled students. Video surveillance was not specifically mentioned in parental consents for the following reasons: a) identities of individuals could not be recognized in the video surveillance due to limited resolution of the footage; b) the cameras were placed in public locations; and c) there was concern that explicitly publicizing video surveillance to parents could amplify student reactivity to the surveillance. It should be noted that both video surveillance and in-person observation captured non-target behaviors at times, such as students fighting or urinating/defecating outside the latrines. Teachers and school administrators were informed that the cameras could not be used to monitor illicit behavior since students were not identifiable in the footage. Ethical approval for the project and all consent procedures described above (including not informing parents of video surveillance) was obtained from the Stanford University Institutional Review Board (PR#: 19143) and the Kenya Medical Research Institute (KEMRI) Scientific and Ethical Research Committees (No. 1840).

## Results

A total of 5,510 student toileting events were captured by in-person observation during 46 unique school-days at sanitizer intervention schools and 51 unique school-days at soap and water intervention schools. Video surveillance captured 2760 toileting events over 21 school-days at sanitizer schools and 20 school-days at soap and water schools. Overall, hand cleaning rates (with water only, soap, and/or sanitizer) captured by video observation were higher (64%) than hand cleaning rates documented during in-person observation (56%); however, the rates were not significantly different when controlling for the individual school at which the data were collected (Table 1). Trends were similar for hand cleaning rates with a product (soap or sanitizer) (Table 1). When stratified by intervention, video surveillance and in-person observation captured similar hand cleaning rates at sanitizer schools (82–84%, RR = 0.99, 95% CI [0.92–1.06]) (Table 1). At soap intervention schools, video surveillance captured significantly higher rates of hand cleaning (42%) as compared to in-person observation data (37%) (RR = 1.14, 95% CI [1.03–1.26]) (Table 1).

**Table 1:** Student hand cleaning rates (% of toileting events) and duration captured by in-person structured observation *versus* video observation.

	Video surveillance	In-person observation	
	All hand cleaning (with or without product) [%, N]		RR (95% CI)φ
All schools	64%, N = 2760	56%, N = 5510	1.04 (0.98–1.12)
Sanitizer schools	82%, N = 1474	84%, N = 2130	0.99 (0.92–1.06)
Soap schools	44%, N = 1286	38%, N = 3380	1.16 (1.05–1.28)*
	Cleaned hands with soap or sanitizer [%, N]		RR (95% CI)φ
All schools	63%, N = 2760	55%, N = 5510	1.04 (0.98–1.10)
Sanitizer schools	82%, N = 1474	84%, N = 2130	0.99 (0.92–1.06)
Soap schools	42%, N = 1286	37%, N = 3380	1.14 (1.03–1.26)*
	Mean duration of hand cleaning in seconds [mean (SD), N]		p-value (t-test)
All schools	49 (44), N = 515	30 (24), N = 2622	P<0.001
Sanitizer schools	34 (26), N = 310	21 (9), N = 1676	P<0.001
Soap schools	71 (55), N = 205	47 (33), N = 946	P<0.001

*p<0.05.

φ Rate-ratios (RR) and p-values reported for Poisson regression analysis of rate differences between video and in-person data, while controlling for the individual school at which the data were collected.

Both methods found that female students cleaned their hands slightly more often than boys; the hand cleaning rate after toileting was 4% higher among girls according to video surveillance (p = 0.02) and 3% higher according to in-person observation (p = 0.01). Average hand cleaning time recorded during video observation was significantly higher (49 seconds, SD 44, N = 515 hand cleaning events) than that recorded during in-person observation (30 seconds, SD 24, N = 2622 hand cleaning events; p<0.01). Both video observation and in-person observation demonstrated longer hand cleaning times for handwashing with soap than rubbing with sanitizer (Table 1).

In-person observation and video surveillance data were directly matched and compared for 22 school-days, each with 3-hours of observation overlap (13 school-days at soap intervention schools, 9 school-days at sanitizer intervention schools). The absolute difference in mean hand cleaning rates captured by in-person observation *versus* video observation was not significant (difference  = 0.04, p = 0.74, N = 44 school-days). Similar results were obtained for comparisons of hand cleaning rates with soap (difference  = 0.03, p = 0.81, N = 44 school-days) and with sanitizer (difference  = 0.00, p = 0.99, N = 44 school-days). However, the mean number of toileting events captured by in-person observation over a 3-hour period was significantly higher than that captured by video observation (mean difference  = 25, p = 0.02, N = 44).

### Reactivity to video surveillance

Overall, in-person observation data collected with and without concurrent video surveillance show significantly higher hand cleaning rates during video surveillance periods (RR = 1.11, 95% CI 1.03–1.20, Table 2). Students at soap intervention schools were 1.3-fold more likely to wash their hands during concurrent video surveillance when compared with periods of in-person observation alone (RR = 1.28, 95% CI 1.14–1.43). By contrast, students at sanitizer intervention schools were not significantly more likely to clean their hands during simultaneous video and in-person observation (RR = 0.99, 95% CI 0.89–1.11, Table 2). Video footage analysts noted instances of students staring directly at cameras while hand cleaning, and documented students pointing and gesturing towards the cameras.

**Table 2:** Hand cleaning rates (% of toileting events) captured by video surveillance data (left), and by in-person observation (right).

	Video surveillance data			In-person observation data		
	Simultaneous monitoring [%, N_V,S_]	Video surveillance only [%, N_V,I_]	RR (95% CI)φ	Simultaneous monitoring [%, N_P,S_]	In-person observation only [%, N_P,I_]	RR (95% CI)φ
All schools	65%, N = 1563	64%, N = 1197	1.08 (0.98–1.19)	60%, N = 1581	54%, N = 3929	1.11 (1.03–1.20)*
Sanitizer schools	83%, N = 738	81%, N = 736	1.02 (0.91–1.14)	84%, N = 593	83%, N = 1537	0.99 (0.89–1.11)
Soap schools	47%, N = 825	39%, N = 461	1.22 (1.02–1.46)*	45%, N = 988	36%, N = 2392	1.28 (1.14–1.43)*

Simultaneous monitoring refers periods when in-person observation and video surveillance were conducted concurrently.

*p<0.05.

φ Rate-ratios (RR) and p-values reported for Poisson regression analysis of rate differences between simultaneous and independent monitoring, while controlling for the individual school at which the data were collected.

During in-depth interviews, several teachers mentioned that video surveillance may have served as a reminder for students to practice hand cleaning, and as a source of motivation for teachers to ensure compliance. One teacher at a sanitizer intervention school stated “once a camera is put in place, it acts as a spy so even if you tend to forget [you] will see it and wash your hands.” A teacher at a soap intervention school said, “you know when there is supervision you cannot be lazy. When the camera was there we made sure there was water in the tank.” A few teachers thought the camera did not affect behavior long-term; one teacher stated: “even when [the camera] was not there it had become a routine for people to wash their hands.”

### Reactivity to in-person observation

Overall, hand cleaning rates were higher during concurrent video and in-person observation compared to during video surveillance alone, although the difference was not statistically significant (RR = 1.08, 95% CI 0.98–1.19, Table 2). Students at soap intervention schools were significantly more likely to wash their hands during concurrent video and in-person observation (RR = 1.22, 95% CI 1.02–1.46); for students at sanitizer intervention schools no significant difference was found (RR = 1.02, 95% CI 0.91–1.14, Table 2). On those days with concurrent in-person observation and video surveillance, hand cleaning rates were lowest prior to the arrival of the field observer (55%), after which they increased by about 10 percentage points during in-person observation (Table 3).

**Table 3:** Average rate of hand cleaning (% of toileting events) captured by video surveillance preceding and during in-person observation (days with concurrent video/in-person observation only).

Hand cleaning, as observed by video surveillance	Preceding in-person observation, N = 368	During in-person observation, N = 1022
All hand cleaning (with or without product)	55%	66%
Hand cleaning with soap or sanitizer	54%	65%

The majority of teachers interviewed mentioned that the presence of study field staff at hand cleaning stations may have improved hand hygiene behavior by students. One teacher noted, “by you sitting there and observing made it easier for me because you helped me watch [the students].” It was noted that the influence of in-person observation was greater at the beginning of the study. One teacher said “with time we got used to you and also to the sanitizer,” while another noted, “the good thing is even after you leave we will still clean our hands as now it is a habit.”

### Peer influence

**Figure 4:**
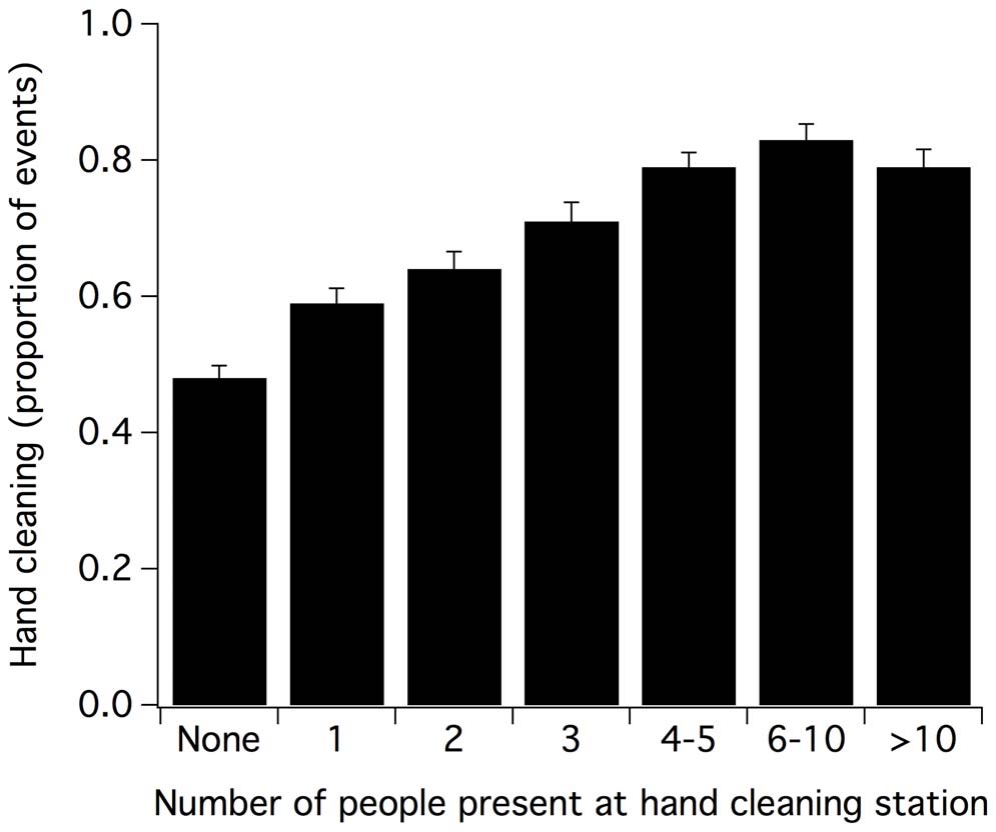
Proportion of student toileting events followed by hand cleaning, shown by number of other people present at the hand cleaning station (visible within the camera view frame). Error bars show standard error of the mean.

Only one student was visible within the camera view frame in 28% of toilet use events captured by video surveillance (N = 2811). Overall, when students were alone at a hand cleaning station, hand cleaning rates averaged 48%, compared to 71% when at least one other student was present (Table 4). At sanitizer schools, students were significantly more likely to clean their hands when one or more other students were viewed near the hand cleaning station (RR = 1.18, 95% CI: 1.02–1.37). This trend was not statistically significant at soap intervention schools (Table 4). Hand cleaning rates showed an overall trend of increasing as the number of other people present at hand cleaning stations increased, with the exception of a slight decrease in hand cleaning when greater than 10 subjects were observed (Figure 4).

**Table 4:** Hand cleaning rates (% of toileting events) when the subject was observed to be alone in the video frame *versus* when other students were present in the frame, as captured by video surveillance.

	Subject alone in video frame, N = 768	Two or more individuals in video frame, N = 1991	RR (95% CI)
All data	48%	71%	1.14 (1.01–1.28)*
Sanitizer schools	71%	85%	1.18 (1.02–1.37)*
Soap schools	32%	51%	1.08 (0.89–1.30)

Video coders noted the following scenarios when more than one person was present at the hand cleaning station: 1) Teachers instructing groups of students on proper hand cleaning technique; 2) Older students assisting younger students (*e.g*. lifting young students up to reach wall dispenser, placing product on hands); 3) Students reminding other students to clean hands and/or demonstrating proper technique; 4) Groups of students engaged in conflict over access to hand cleaning materials (*e.g*. pushing, hitting); 5) Students being called away from hand cleaning stations by teachers to attend class.

## Discussion

Analysis of video surveillance footage captured by cameras placed at shared school latrines yielded similar rates of hand cleaning by students post-toileting to those found by in-person structured observation during the same time period. This work suggests that video surveillance of hand hygiene behavior yields valid results that are comparable to in-person observation in a resource-constrained setting.

We found evidence of student reactivity to both video surveillance and in-person observation, in the form of higher hand cleaning rates at handwashing with soap intervention schools. Reactivity was not detected at sanitizer intervention schools; this may be explained by the fact that hand cleaning rates with sanitizer were over 80%, leaving less room for compliance rates to shift upwards. Reactivity to simultaneous video and in-person observation increased handwashing rates by similar magnitudes (28% and 22%, respectively). These data should be interpreted as the marginal impact of each observation method on behavior when the other method is also in effect, as reactivity to each method could be higher when compared to a period during which no observation is occurring. Reactivity measured in this study was less than the 35% increase in hand cleaning frequency due to structured observation found by Ram and colleagues (2010) among female caregivers of young children [5]. Previous studies documenting reactivity to in-person observation have been principally conducted with adult subjects [17]–[19]. It is possible that students may be less likely than adults to exhibit reactivity to in-person observation, as they are accustomed to being watched by teachers. Examining changes in reactivity over time (*e.g*. decreased reactivity as the novelty of observation wore off) for each method was outside the scope of this study, and would be a valuable area for future research.

The presence of at least one other person at the hand cleaning station increased student hand cleaning rates by 14% among all schools, indicating that peer influence can significantly impact student hand cleaning compliance. Reaction to peer presence has previously been documented to increase hand hygiene compliance among adults. A previous study found that female students cleaned their hands 91% of the time after using the bathroom when someone else was present, compared to 55% of the time when they were alone in the sink area [20]. We also found that hand cleaning rates increased proportionally with the number of additional people observed in the immediate vicinity of the hand cleaning station, up to a threshold of 10 people (Figure 4). Higher rates of hand cleaning in groups indicate that the intervention established hand cleaning as a social norm among the students, an important motivation for hand cleaning behavior [21]. It is also possible that the presence of other individuals at the hand cleaning station served as a reminder or cue, whether conscious or unconscious, for a student to stop and hand clean. Video analysts recorded a number of phenomena that could explain the drop in hand cleaning rates during crowding events (>10 people), including students being unwilling to wait in queues to use the hand cleaning facilities, physical conflicts between students, and teachers instructing students to go to class.

The positive influence of peer presence on student hand hygiene behavior observed in this study has key implications for the design and implementation of future hand hygiene programs in schools. Placement of hand cleaning materials in public locations, along with the scheduling of specific times for bathroom breaks between classes, could significantly improve student hand hygiene compliance rates. Strategies to instill hand hygiene as a social norm may also be effective, such as designating specific students to be hand hygiene “champions,” or the formation of student clubs to demonstrate and promote hand hygiene to their classmates [22].

Some disadvantages to using video surveillance were evident. Cameras had to be padlocked during the day and removed nightly to avoid theft and vandalism. Minimal available space and flimsy school walls made installation and stable positioning of the camera difficult. On some occasions, cameras were knocked or fell into positions that did not capture the full view of the latrine entrance and hand cleaning station. Notably, it was infeasible to utilize cameras to record hand cleaning at control schools in this study, because one school did not keep its hand cleaning materials (i.e. portable basin and soap) in a specific place. Video surveillance is thus best suited for monitoring compliance at fixed hand cleaning stations.

We found that video surveillance has several potential advantages as an observational method. First, video surveillance significantly reduced the amount of local staff time necessary for data collection. The cameras had motion sensors to trigger recording only when movement was detected at hand cleaning stations, thus eliminating staff time spent observing the hand cleaning station when latrines were not in use. Moreover, video footage could be analyzed at increased speeds. Video surveillance also allowed off-site research staff to view intervention compliance directly, to replay footage to capture additional information, and to witness unexpected reasons for non-compliance. Video surveillance is a useful method for monitoring hand hygiene behavior that enables rapid synthesis of rich behavioral data.
